# Proceedings of the Lippe Society

**Published:** 1889-03

**Authors:** George H. Clark

**Affiliations:** Secretary


### PROCEEDINGS OF THE LIPPE SOCIETY.

The one hundred and twenty-ninth meeting of the Lippe Society was held on Tuesday evening, February 12th. There were present Drs. C. Carleton Smith, M. Preston, F. Powel, Lee, W. R. Powel, Farley, Clausen, James, and Clark. Dr. Smith occupied the chair. The minutes of the previous meeting were read and approved. Dr. Lee then read a communication from Dr. Berridge, of London, with some interesting cases, showing the proper method of selecting the remedy, for which the thanks of the members were tendered Dr. Berridge.

Dr. Clausen then read a paper (see p. 162) on Theridion, with a prelude upon the proper method of a Society's work and its decorum. After the reading, Dr. Clausen said he was very much interested in Theridion, and wished the members would give him any verifications they had noted. Dr. Smith had repeatedly verified it in vertigo on closing the eyes, and found one dose of the CM sufficient. Dr. Preston had often used it for violent frontal headache, with heavy, dull pressure behind the eyes. Dr. Lee called attention to the vomiting when closing the eyes. Dr. Clark spoke of the peculiar cough, which is a frequent, convulsive cough, during which the head is spasmodically jerked forward, and the knees jerked up toward abdomen. Dr. Farley said that with Therid.$^{30}$ he was always able to make happy a woman who suffered terribly from symptoms of spinal irritation with severe headache. The indication: she could not bear the least noise, and the jar of the foot on the floor was so aggravating that it made her cry out.

Dr. Clausen—Bell. has aggravation from jarring, and even touching the bed, particularly in uterine affections.

Dr. Farley—Bell. has the symptoms of congestion; Therid. extreme nervous symptoms. It is similar to Sulph. as an intercurrent in the psoric diathesis.

Dr. Clausen—Dr. Baruch ascribes to it marked antipsoric power.

Owing to the unavoidable absence of Dr. Still, who had been appointed to read a paper on diphtheria, that subject was continued for the next meeting after Dr. Lee read some notes of Drs. Hering and P. P. Wells upon the subject.

Dr. Farley then read notes of two or three difficult cases, and asked advice. The cases were studied and suggestions for the remedy given.

Dr. Lee then read the cases sent by Dr. Berridge. Dr. Preston, commenting upon the Lactuca case, said that some years ago he had been able to make a cure with Lactuca in a woman who had apparent disease of the pancreas. Patient could not retain food; and there was much pain in region of the stomach. From the *Symptomen Codex* he learned the value of Lactuca.

Dr. James then said that within a short time he had seen several cases which strongly confirmed what Dr. Lippe had frequently told him about the difficulty in getting the important, peculiar symptoms from some patients. How they will ignore the facts, or so answer that one is not able to get at the proper remedy. Dr. James related the two following cases in his own experience, illustrating this point: A young lady was seized with violent pains in the muscles in the region of the right hip. The indications were very obscure, and no remedy could be given. It was not until the third visit that the symptoms leading to the choice of the remedy could be obtained. On entering the room the doctor found the patient lying upon the painful side. On inquiring why she lay that way, she answered that it was impossible to lie in any other position. She added that she was worse from the slightest motion. Here were clear indications for Bryonia, which was accordingly given, and the pain began to improve immediately. That night she had a profuse perspiration, and the next morning she was well. The perspiration following the administration of Bryonia was a favorite indication with the venerable Dr. Lippe that Bryonia had been correctly prescribed. Dr. Lippe has spoken to Dr. James many times of this sweat, and insisted upon the certainty of its occurrence when Bryonia was the true simillimum.

The second case spoken of by Dr. James was a case of consumption occurring in one of Dr. Lippe's patients. One afternoon a week ago the patient awoke from his afternoon nap with sense of suffocation. The cough had entirely disappeared and a terrible struggle for breath had begun. Every inspiration was followed by a groaning expiration. The symptoms given by the patient were indefinite, and it seemed impossible to select the remedy. Several hours passed without relief when accidentally the sister of the patient spoke of his being *wakened from his afternoon nap* by the dyspnœa, and that in great haste they had *cut away the collar* from his neck because *he could not bear the slightest pressure upon the neck.* The instant the doctor heard these statements, he gave Lachesis[5m] (B. & T.) in water, and great relief quickly followed. In both these cases valuable time had been lost by the neglect of the patient to give the symptoms at first. The severe cross-questioning only seemed to puzzle the patient and attendants, and baffle the doctor in getting the correct indications.

Cases for consultation were then called, and Dr. Farley offered the following: Child æt. seven months. Eructates mouthfuls of food, and a sour, acrid, watery substance. Copious and frequent belching, which aggravates. Thirst for large draughts; worse in evening. Tongue coated yellowish-brown. Sour smelling, corrosive stool. Kreosote and Ferrum were suggested for study.

Woman æt. fifty-five years. Angina faucium for twenty-nine years. Constant aching pain in roof of mouth, and in post-nasal space, and at bridge of nose. Soreness of the sterno-cleido-mastoid muscle, left side; *worse soon as weather gets cool. No soreness on swallowing.* "Blind spells" come on occasionally—that is, temporary dimness of vision, as though a heavy, dark veil were before eyes. Aching, tired feeling over eyes in morning; passes off after she stirs around awhile. Very fond of salt; often excessive thirst. *Suppressed intermittent fever.* Suppuration in throat occasionally. Raw feeling from contact with *cool* air, and highly-seasoned food. Soreness disappears for a time only to reappear. Sometimes talking hurts much, and sometime only a little. Therid., Lachesis, and Lac caninum were recommended as bearing on the case.

Dr. Clark then suggested that the Lippe Society celebrate Hahnemann's birthday by a banquet. The suggestion was approved. The Society then adjourned.

GEORGE H. CLARK, *Secretary.*

